# Post-Kala-Azar Dermal Leishmaniasis: A Paradigm of Paradoxical Immune Reconstitution Syndrome in Non-HIV/AIDS Patients

**DOI:** 10.1155/2013/275253

**Published:** 2013-03-24

**Authors:** Eltahir Awad Gasim Khalil, Selma Abdelmoneim Khidir, Ahmed Mudawi Musa, Brema Younis Musa, Mona Elfaki Eltahir Elfaki, Abdelgadir Mohamed Yousif Elkadaru, Edward Zijlstra, Ahmed Mohamed El-Hassan

**Affiliations:** ^1^The Leishmaniasis Research Group, Sudan; ^2^Institute of Endemic Diseases, University of Khartoum, P.O. Box 45235, 11111 Khartoum, Sudan; ^3^The Central Laboratory, Ministry of Science & Communications, 7099 Khartoum, Sudan; ^4^Tropical Diseases Hospital, Omdurman, Sudan

## Abstract

Visceral leishmaniasis (VL) is a parasitic disease characterized by immune suppression. Successful treatment is usually followed by immune reconstitution and a dermatosis called post-Kala-azar dermal leishmaniasis (PKDL). Recently, PKDL was described as one of the immune reconstitution syndromes (IRISs) in HIV/VL patients on HAART. This study aimed to present PKDL as a typical example of paradoxical IRIS in non-HIV/AIDS individuals. Published and new data on the pathogenesis and healing of PKDL was reviewed and presented. The data suggested that PKDL is a typical example of paradoxical IRIS, being a new disease entity that follows VL successful treatment and immune recovery. PKDL lesions are immune inflammatory in nature with granuloma, adequate response to immunochemotherapy, and an ensuing hypersensitivity reaction, the leishmanin skin test (LST). The data also suggested that the cytokine patterns of PKDL pathogenesis and healing are probably as follows: an active disease state dominated by IL-10 followed by spontaneous/treatment-induced IL-12 priming, IL-2 stimulation, and INF-**γ** production. INF-**γ**-activated macrophages eliminate the *Leishmania* parasites/antigen to be followed by LST conversion and healing. In conclusion, PKDL is a typical example of paradoxical IRIS in non-HIV/AIDS individuals with anti-inflammatory cytokine patterns that are superseded by treatment-induced proinflammatory cytokines and lesions healing.

## 1. Introduction

*L. donovani *infections are widely prevalent in East Africa and the Indian subcontinent manifesting as a wide spectrum of clinical phenotypes ranging from subclinical infections to a potentially fatal visceral disease. Visceral leishmaniasis (VL) is a parasitic febrile illness with a transient immune suppression state with leucopenia and increased IL-10 secretion [1–4]. In the HIV/AIDS era, VL is considered an opportunistic infection as evidenced by emergence of HIV/VL coinfections [5–10]. VL successful treatment is characterized by improvement of the leucopenia with a decline in CD4+ T cells and conversion in the leishmanin skin test (LST), a probable immunity surrogate marker. LST conversion probably indicates (re) constitution of transiently lost cell-mediated immunity against *Leishmania* antigens [1, 11–16]. In VL, IL-4 stimulation with IL-10 overproduction leads to reciprocal inhibition of INF-*γ* production and polyclonal B-cells stimulation (Th2 immune response) [17–20].

More than fifty percent of successfully treated Sudanese VL patients develop an inflammatory skin rash, called postkala-azar dermal leishmaniasis (PKDL). A number of hypotheses have been put forward to explain the aetiology of PKDL: undertreatment, UVB light exposure, and ethnicity [1, 21–23]. LST conversion, high plasma and skin IL-10, high plasma levels of C-reactive protein and high TGF-*β* during VL predict development, progression and severity of PKDL [20, 24, 25]. PBMCs and skin immune responses of VL/PKDL patients are dichotomous. It start as Th2 immune response in VL patients, pass through a mixed Th1/Th2 stage to be followed by a pure Th1 response in cured patients [26, 27]. The majority of PKDL patients heal spontaneously, with persistence in 15% with chronic lesions that are probably a reservoir of infection [22, 28, 29]. Sodium stibogluconate (SSG), liposomal amphotericin B (Ambisome), and immunochemotherapy (SSG in combination with alum-precipitated autoclaved *L. major *vaccine) are available treatment modalities [22, 30, 31].

Immune reconstitution inflammatory syndrome (IRIS) is a well-documented phenomenon that follows immune reconstitution in HIV patients who have recently started HAART. It is a stereotyped immune inflammatory state that is characterized by transient worsening or appearance of new symptoms/signs following successful treatment. IRIS has been described in patients with parasitic, bacterial, viral and autoimmune diseases [32–38]. CD4+ counts and preexisting opportunistic infection are reliable predictors of IRIS development [39–41]. The immune pathology of IRIS is largely determined by the infecting organisms where CD8+ T cells dominate lesions of viral origins; granulomatous inflammation usually dominates IRIS of fungi, protozoa, and *mycobacterial *conditions [42–50]. IRIS manifests when there is an abrupt shift from an anti-inflammatory and immuno-suppressive state mediated by TNF-*α* and IL-10 to a pro-inflammatory state mediated by variable levels of IL-2, IL-12 and IFN-*γ* [38, 51–55]. An increase in CD4+/CD 8+ cells coupled with a reduction in T_reg_ cells and an exaggerated cytokines response lead to initiation and progression of IRIS [56–61]. 

Recently, PKDL was reported as an IRIS phenomenon from Africa and Europe in HIV/AIDS/VL co-infected patients [62, 63].

This study aimed to present PKDL as a form of paradoxical IRIS in non-HIV/AIDS patients with plethora of cytokines production, granuloma formation, and delayed-type skin hypersensitivity reaction (LST) without activation of existing opportunistic infection.

## 2. Materials and Methods

Archived supernatant samples from in vitro stimulated PBMCs of thirty PKDL patients were selected from the samples bank of the Institute of Endemic Diseases, University of Khartoum. PKDL patients were enrolled in an immunochemotherapy study and were randomized to two study arms: patients in group I received four intradermal doses of 100 *μ*g alum-precipitated ALM + BCG (BCG 1/10th usual vaccine dose)/weekly plus daily sodium stibogluconate (SSG). Patients in group II received daily SSG plus four doses of the vaccine diluent (placebo). SSG was given intramuscularly/intravenously at a standard dose of 20 mg/Kg body weight/day [27].

The study protocol was reviewed and passed by the Ethics and Scientific Committees of the Institute of Endemic Diseases, University of Khartoum and the Ethics Committee of the Federal Ministry of Health, Khartoum. Samples were from patients who consented previously for the storage and use of their samples for further future testing. Patients were enrolled in the study based on specific inclusion and exclusion criteria as described previously [27].

PKDL patients were subjected to physical examination, pregnancy testing, DAT/HIV serological test, skin biopsy, haematological and chemical tests, LST, ECG, and cytokines at screening (D2), during treatment (D0, D7, D14, and D21) and follow up (D30, D40, D60, and D90) periods.

PBMCs were harvested using the density gradient centrifugation and were counted using Trypan blue exclusion technique with a haemocytometer. PBMCs cultures were stimulated with soluble *L. donovani* antigen; phytohemagglutinin (PHA) as a positive contol, a third well was left without antigen or mitogen as a negative control. Supernatants were stored at −80°C for later analysis. IL-10 and IFN-*γ* were measured using double sandwich technique as per manufacturer's leaf-lets (R&D Systems, Germany). Results were previously reported [27]. IL-2 and IL-12 were measured using commercial kits (R&D Systems, Germany).

## 3. Results and Discussion

### 3.1. Group I (Alum/ALM Vaccine/BCG + SSG)

Total number of patients enrolled in this group was fifteen. 

*Five patients* (data not shown on table) had low to undetectable IL-2, IL12, and IFN-*γ* levels on D-2 (screening) and D60 in response to soluble *L. donovani* antigen (sLA). The leishmanin skin tests (LST) changed significantly from nonreactive (induration = 00 mm) on D-2 to reactive (induration = 8.0 ± 1.4 mm) on D60. All five patients healed completely by D60 of followup. These patients most probably passed IL-12 priming, IL-2 stimulation and IFN-*γ* secretion (pro-inflammatory stage), and are now in leishmanin skin reactivity (memory) and lesion healing. 

*Four patients* [nos. 107, 118, 116 and 129] showed low levels of IL-2 and IL-12 on D-2 and D60. Their IFN-*γ* was high on D-2 but dropped significantly on D60 in response to soluble *L. donovani* antigen (sLA). Three (3/4, 75%) were LST reactive on D-2 and remained the same on D60. The fourth patient converted in LST on D60 of follow up. The skin lesions of the four patients healed completely by D60. It is probable that these patients passed IL-12 priming/IL-2 stimulation and were seen in IFN-*γ* secretion (pro-inflammatory stage) where activated macrophages eliminated the *Leishmania* parasite/antigen paving the way for immune recovery (LST conversion) and healing (Table 1).

*Three patients* [nos. 121, 123 and 102] had low IL-2 and IL-12 on D-2 and D60, the IFN-*γ* levels increased significantly on D60 compared to screening levels in response to soluble *L. donovani* antigen (sLA) and were accompanied by LST conversion. All three patients healed completely by D60. These patients already passed IL-12 priming/IL-2 stimulation when screened and were slowly creeping into IFN-*γ* secretion (pro-inflammatory stage) and LST conversion and healing. Alternatively, these patients probably passed the IL-12 priming when screened and were not yet in the IL-2 stimulation. IL-2 stimulation was probably initiated later when immune-chemotherapy was started, leading to increased IFN-*γ* secretion and LST conversion and healing (Table 1).

*Two patients* [nos. 110, 105] had high IL-2 levels on D-2 that significantly drop on D60, while their IL-12 was low at both dates. Their IFN-*γ* levels dropped significantly on D60 compared to their D-2 levels in response to soluble *L. donovani* antigen (sLA). Both patients converted in LST by D60 of follow up. One patient healed while the other did not and had to receive Ambisome treatment for the lesions to heal. These patients passed the IL-12 priming stage and were seen at the IL-2 stimulation/IFN-*γ* secretion stage (pro-inflammatory stage) that is followed by IFN-*γ* reduction and LST conversion (Table 1).

*One patient* [nos. 104] had high IL-2 on D-2 that increased significantly on D60, while IL-12 was low through the follow up period. The IFN-*γ* level was markedly high on D-2 and dropped significantly on D60 with LST conversion. This patient completely healed on D60 of follow up. This patient was probably in IL-2 stimulation/IFN-*γ* secretion with overlap of LST conversion and healing (Table 1).

It is evident that all patients in the Alum/ALM vaccine + BCG group passed the anti-inflammatory stage when screened (low to absent IL-10), some were in the pro-inflammatory (IL-2/IL-12/IFN-*γ* secretion) with progression to LST conversion and healing by D60. Their LST mean induration was 8.3 ± 1.7 mm (median= 7 mm) and was significantly different from D-2 LST induration (*P* < 0.000). Patients passed a probably short lived IL-12 priming state when screened. Some were in the IL-2 stimulation/IFN-*γ* secretion/LST conversion/healing stage, while others had passed the IL-2 stimulation stage and were in IFN-*γ*/LST conversion/healing. Majority of patients (14/15; 93.3%) healed completely by D60. The SSG vaccine combination appears to be effective in eliminating the *Leishmania* parasite/antigen relieving the immune paresis that was preventing healing in these patients.

IL-10 levels were uniformly low in response to soluble *L. donovani* antigen (sLA) in all screening and follow up samples in all patients. This confirms that patients overcame the anti-inflammatory stage. The progression from an anti-inflammatory to a pro-inflammatory stage has been reported previously as a prerequisite for the development of IRIS in HIV/TB co-infected patients on HAART [51–54].

### 3.2. Group II (SSG + Vaccine Diluent)

Total number of patients enrolled in this arm was fifteen.

*Seven patients* [nos. 109, 106, 103, 101, 130, 115 and 127] had low IL-2 and low IL-12 on D-2 with similar levels on D60 except for one patient [no. 106] who showed an increase in IL-2, a drop in IFN-*γ* with no LST conversion, and a complete healing by D60. Six patients of the above [6/7, 85.7%; nos. 109, 106, 103, 101, 130, and 115] had high IFN-*γ* levels on D-2, that is, in a pro-inflammatory stage. Five patients [5/7, 71.4%; nos. 109, 106, 103, 101, 130] showed marked to moderate drop in D-2 IFN-*γ* levels, while the other two [2/7, 28.6%; 115, 127] had a significant increase in IFN-*γ* on D60, that is, progressive IFN-*γ* secretion (anti-inflammatory stage). The majority of the seven patients [87.5%] were LST non-reactive on D-2 compared to 62.5% on D60. The majority (85.7%) of these patients healed completely by D60. It is probable that most of these patients passed the IL12 priming (?Transient IL-12 priming) and the IL-2 stimulation and were in the IFN-*γ* secretion stage when seen on D-2. On D60, some of these patients continued in IFN-*γ* secretion, while others dropped their IFN-*γ* with consequent LST conversion and healing (Table 1).

*Six patients* had low to undetectable IL-2, IL-12, and IFN-*γ* in response to soluble *L. donovani* antigen (sLA) on D-2with similar levels on D60. The majority (5/6; 83%) were LST non-reactive on D-2 and remained the same on D60 with only one healed patient. Another patient (1/6; 17%) who was strongly LST reactive on D-2 and remained the same on D60; he progressed to complete healing. The healed two patients probably passed the IL-12 priming, the IL-2 stimulation and IFN-*γ* production and were in LST conversion status with complete cure. The nonhealing 4 patients were probably in an “immune paresis” state and were not able to mount IL-12 priming, IL-2, and IFN-*γ* production/LST conversion and so exhibited no healing (?high leishmania antigen load). These patients healed completely with Ambisome treatment. Failure to progress to IL-2 priming and IL-12/IFN-*γ* secretion in these patients probably indicates a degree of parasite unresponsiveness to SSG leading to persistence of *Leishmania* parasite/antigen and the observed immune paresis as evidenced by lack of LST conversion. Ambisome was successful in overcoming the parasite unresponsiveness, which is indicated by lesions healing.

*Patient no. 112* had low IL-12, high IL-2, and high IFN-*γ* and LST non-reactivity on D-2. On D60, the IFN-*γ* and IL-2 drop was accompanied by LST conversion and healing. This patient probably passed IL-12 priming and was in IL-2 stimulation and IFN-*γ* secretion when screened. The IFN-*γ* drop, LST conversion and healing were achieved during the follow up period. This patient findings demonstrate the mirror-image pattern exhibited by IL-2 and IFN-*γ* levels.

*Patient no. 108* had low IL-12, high IL2/IFN-*γ* on D2, with IL-2 remaining high on D60 with a drop in IFN-*γ* level LST conversion and healing. The high level of IL-2 with low IFN-*γ* levels and LST conversion and healing needs explanation!

IL-10 levels in response to soluble *L. donovani* antigen (sLA) were uniformly low in all screening and follow up samples, that is, patients passed the anti-inflammatory state when screened. These patients overcame the anti-inflammatory stage, progressed to a pro-inflammatory in line with previous data on IRIS in HIV co-infected patients on HAART [51–54].

Some of the patients in the SSG/vaccine diluent were in a state of immune paresis (high leishmania antigen load) and were unable to mount an IL-12 priming and IL-2/IFN-*γ* secretion/LST conversion and healing. As suggested previously, a state of parasite SSG-unresponsiveness could be a contributory factor in patients lingering in an immune paresis stage. Ambisome treatment eliminated the parasite with reduction in *Leishmania* antigenic load putting patients on the way to recovery. Others in this group showed the typical natural history of PKDL healing, that is, passing IL-12 priming and were seen in the IL-2/IFN-*γ* secretion that was followed by LST conversion and healing. 

Data from this study showed the clear dichotomy of immune response in PKDL patients as was previously reported [64]. On the other hand, the levels of IL-2 and IFN-*γ* more or less mirror-image each other.

 Case reports from Africa and Europe introduced PKDL as an immune reconstitution phenomenon in HIV/VL co-infected patients at the start of HAART [62, 63]. In this study we attempted to prove that PKDL is an IRIS phenomenon that develops in VL patients who go through a transient stage of immune depression. PKDL develops as a new disease entity with symptoms and signs that are mainly of skin origin and that are different from VL. Cured VL patients become immune competent as evidenced by conversion of the LST at six months after treatment, around the same time of PKDL development [29]. Healing of PKDL lesions is a function of a change of immune responses from a mixed Th1/Th2 state (anti-inflammatory) to a pure Th1 one (pro-inflammatory). Healing of skin lesions starts at the cellular level by antigen presentation, followed by IL-12 priming, and IL-2 secretion which facilitates expansion of the Th1 population and IFN-*γ* and TNF-*β* secretion. It is logical to assume that the sequence of events in PKDL healing is as follows: drug-induced parasite killing, antigen load reduction, IL-12 priming followed by IL-2 secretion that in turn induces IFN-*γ* secretion which augments the killing potential of the macrophages with production of nitric oxide and reactive oxygen intermediates. Eventually, inflammation decreases and healing occurs with production of memory cells and a lifelong LST reactivity status. The data also points to the fact that the cytokine patterns of PKDL healing are stereotyped and the differences between drug-treated (SSG; Ambisome), and immunochemotherapy-induced patterns are only quantitative. IL-2 is most probably the initiating cytokine for the healing process in PKDL, a finding that may have future therapeutic implications.

*In conclusion*, PKDL is a form of paradoxical IRIS that emerges as a new disease entity following successful VL treatment and immune recovery. PKDL skin lesions are immune inflammatory in nature and respond adequately to immuno-chemotherapy. Like IRIS, PKDL is an immune-mediated phenomenon with increased activation from antigenic exposure, granuloma formation, and skin hypersensitivity reaction (LST conversion). Different Th1 and Th2 cytokines play important roles in PKDL pathogenesis and healing. IL-2 plays a pivotal role in PKDL healing process.

## Figures and Tables

**Table 1:** IL-2, IL-12, INF-*γ*, and IL-10 levels and LST induration (mm) in some patients in the study.

ID	Day 2					Day 60					Treatment outcome
	IL-2	IL-12	INF-*γ*	IL-10	LST	IL-2	IL-12	INF-*γ*	IL-10	LST	
110*	**716**	00	**2505**	16	00	135	4.8	**438**	22	**07**	Not healed
107*	55	2.9	**2428**	21	03	80	07	**865**	08	**07**	Healed
104*	**1220**	4.8	**2349**	38	03	**1530**	18	**1342**	47	**11**	Healed
116*	00	00	**1016**	33	08	34	03	**968**	81	**10**	Healed
105*	**150**	00	**720**	00	00	30	18	27	48	**06**	Healed
121*	00	08	00	00	00	20	04	**531**	22	**07**	Healed
123*	00	00	101	04	00	00	06	**209**	09	**10**	Healed
118*	00	02	**427**	12	08	00	4.8	85	36	**09**	Healed
129*	00	00	**342**	19	06	15	16.5	**270**	40	**11**	Healed
102*	00	1.8	74	00	00	42	4.8	**874**	35	**07**	Healed
112	**970**	06	**2584**	345	03	45	00	151	08	**08**	Healed
109	00	3.6	**2584**	09	00	25	05	**626**	17	**08**	Healed
108	155	00	**2271**	19	00	185	00	**1231**	00	**05**	Healed
106	45	4.8	**2193**	00	00	**420**	10.5	**1342**	29	00	Healed
103	00	3.6	**1732**	00	00	52	10	**1342**	12	**06**	Healed
101	25	18	**1081**	33	00	100	02	**1342**	32	00	Not healed
130	00	1.2	**813**	102	04	00	00	14	20	—	Not healed
115	00	00	**118**	113	**08**	23	18	**704**	62	**12**	Healed
127	00	6.0	00	46	00	00	12.9	**430**	65	**07**	Healed

*Group I patients (SSG + vaccine). IFN-*γ*, IL-2, Il-12, and IL-10 levels are expressed in pictogram/mL; leishmanin skin test (LST) induration is expressed in mm.
